# The Diabetic Cognitive Impairment Score for Early Screening of Cognitive Impairment in Type 2 Diabetes Patients

**DOI:** 10.1155/jdr/8029913

**Published:** 2025-04-16

**Authors:** Shujun Zhang, Xiaoli Shi, Shaolin Zheng, Xiaoli Liang, Fen Wang, Weijie Xu, Xuefeng Yu, Yan Yang

**Affiliations:** ^1^Division of Endocrinology, Department of Internal Medicine, Tongji Hospital, Tongji Medical College, Huazhong University of Science and Technology, Wuhan, Hubei Province, China; ^2^Hubei Clinical Medical Research Center for Endocrinology and Metabolic Diseases, Wuhan, Hubei Province, China; ^3^Branch of National Clinical Research Center for Metabolic Diseases, Wuhan, Hubei Province, China; ^4^Division of Endocrinology, Jingzhou Hospital Traditional Chinese Medicine, Jingzhou, Hubei Province, China; ^5^Division of Endocrinology, Wenchang People's Hospital, Wenchang, Hainan Province, China

**Keywords:** cognitive impairment, DCIS, P-tau181, T2D

## Abstract

**Purpose:** Diabetes has been associated with an excess risk of cognitive impairment. The hyperphosphorylation of tau protein leads to neurodegeneration and is closely related to Type 2 diabetes (T2D). This study aimed to characterize the association between P-tau181 and diabetic cognitive impairment and to develop a nomogram-based score to screen cognitive impairment in T2D patients.

**Methods:** We used a cohort of 379 patients diagnosed with T2D as a training dataset to develop a predictive model. Risk factors associated with cognitive impairment were identified using stepwise multivariate logistic regressive analysis. A nomogram was established by incorporating these risk factors, and the diabetic cognitive impairment score (DCIS) was built and externally validated in another cohort.

**Results:** In the training cohort, patients with cognitive impairment had higher levels of P-tau181 (13.3 [10.5–18.7] vs. 10.0 [8.0–13.0], *p* < 0.001). P-tau181 was negatively correlated with MOCA (*r* = −0.308, *p* < 0.001) and MMSE (*r* = −0.289, *p* < 0.001), and it was independently associated with cognitive impairment in T2D patients (OR, 1.137 [95% CI, 1.080–1.198]; *p* < 0.001). Other independent risk factors of diabetic cognitive impairment included age, education level, and diabetic retinopathy. The DCIS was built by nomogram based on the four risk factors, which had an area under the receiver operating characteristic curve (AUC) of 0.795 (95% CI, 0.751–0.840). The optimal cut-off of DCIS for the diagnosis of cognitive impairment in T2D patients was 139.5, with a sensitivity of 72.9% and a specificity of 75.3%. In the validation cohort, the AUC of DCIS for screening diabetic cognitive impairment was 0.770 (95% CI, 0.716–0.824).

**Conclusions:** P-tau181 was independently associated with diabetic cognitive impairment. The DCIS, based on P-tau181, age, education level, and diabetic retinopathy, is effective to identify cognitive impairment in T2D patients.

## 1. Introduction

Diabetic cognitive impairment generally refers to cognitive impairment in diabetic patients [1]. The main risks of diabetes are acute and chronic complications. Diabetes also significantly affects patients' cognitive function. The World Alzheimer's Disease Report (2015 Edition) shows that approximately 7%–13% of dementia are related to diabetes [2]. Moreover, a recent meta-analysis of 122 studies observed that diabetes conferred a 1.25–1.91-fold excess risk for cognitive impairment and dementia [3]. As a complication of diabetes, cognitive impairment is attracting increasing attention now.

Cognitive impairment includes two main stages: mild cognitive impairment (MCI) and dementia. MCI refers to a disease state in which memory or other cognitive functions progressively decline but do not affect the ability of daily living and do not meet the diagnostic criteria for dementia [4]. MCI is a transitional state between normal cognitive functioning and dementia. Dementia is a syndrome with acquired cognitive impairment as the core feature, which is significant enough to interfere with independent daily functioning [5]. The prevalence of dementia in Type 2 diabetes (T2D) patients is approximately 17.3% [6]. In the general population, the annual conversion rate of MCI to dementia is about 5%–20% [7], and the conversion rate is accelerated in patients with diabetes mellitus [8].

The exact mechanism underlying cognitive dysfunction in diabetes remains unclear. Insulin resistance, a characteristic finding in patients with T2D, is implicated as an essential etiopathogenetic contributor for cognitive dysfunction [9]. Insulin acts on the central nervous system, not only modulating peripheral metabolism, enhancing systemic insulin sensitivity, and inhibiting endogenous glucose production but also regulating cognition. Other hypotheses include structural changes in brain tissue, changes in cerebral blood flow, abnormal metabolism of brain cells, insulin deficiency and impaired insulin signaling pathways, chronic low-grade inflammation, immune dysregulation, mitochondrial dysfunction, and adipokines (such as adiponectin), all of which lead to impaired neural cell structure and function, which eventually affects cognitive function [10–13].

Currently, neuropsychological assessment is the main method for diagnosing cognitive impairment. Commonly used diagnostic evaluation tools include the Mini-Mental State Examination (MMSE), Montreal Cognitive Assessment (MOCA), Clinical Dementia Rating Scale, and Alzheimer's Disease Assessment Scale (ADAS-cog). However, neuropsychological testing is time-consuming (ranging from 3 to 12 h) and requires specialized clinical personnel [14]. Thus, its application in nonneurology departments is greatly limited, leading to a large number of diabetic patients with cognitive impairment not being diagnosed timely. Therefore, it is critical to develop new and simpler screening tools for diabetic cognitive impairment.

In clinical practice, there are few reliable biomarkers to detect cognitive impairment in T2D patients. The hyperphosphorylation of tau protein leads to the formation of neurofibrillary tangles (NFTs) resulting in neurodegeneration and is closely related to T2D [15]. Emerging evidence suggested plasma phosphorylated tau181 (P-tau181) was associated with memory decline and incidence of dementia, which highlights the potential of plasma P-tau181 as a cost-effective and promising tool for rapid diagnosis of cognitive impairment [16–20]. It is also reported that P-tau181 concentrations are increased in T2D patients and significantly correlated with the decrease of gray matter volume, emphasizing a potential association between P-tau181 with diabetic brain injury [21]. But whether P-tau181 plays an important role in identifying cognitive impairment in T2D patient needs to be further clarified.

Therefore, we aimed to characterize the usefulness of P-tau181 for identifying individuals with the risk of diabetic cognitive impairment and to develop and validate a nomogram-based score for screening cognitive impairment in T2D patients.

## 2. Materials and Methods

### 2.1. Study Cohort

For this retrospective, case-control study, we enrolled patients diagnosed with T2D from Tongji Hospital between December 6, 2020, and January 10, 2022 (*n* = 379). The study cohort was used as a training dataset to develop the predictive model. To test the reproducibility of the prediction model, the model was then validated using an independent cohort obtained from the Optical Valley Branch of Tongji Hospital between May 10, 2022, and November 2, 2022 (*n* = 288). The criteria for inclusion were as follows: (1) patients ≥ 40 years and (2) diagnosis of T2D. The exclusion criteria were as follows: (1) pregnant women, (2) patients with psychological or mental disorders, (3) patients with a history of drug or alcohol abuse, and (4) patients unable to complete the psychological scale screening (hearing and speech disorders). The flowchart is presented in Figure S1.

This study conformed to the standards of medical ethics and was approved by the Ethical Committee of Tongji Hospital (approval no.: TJ-IRB20200703). Written informed consent was obtained from all patients.

### 2.2. Diagnosis of Cognitive Impairment

We adopted neuropsychological assessments (MMSE and MOCA) to evaluate cognitive function. The MMSE focuses on screening for overall cognitive function (time and place orientation, calculation, memory, language ability, attention, and visuospatial ability). It includes 30 questions with a total score of 30 points [22]. The MOCA, an assessment tool, is used for rapid screening for MCI. It includes 11 items distributed in eight cognitive domains including attention and concentration, executive function, memory, language, visual structure skills, abstract thinking, calculation, and orientation [23]. There are standard questionnaires for both tests.

We adopted the general cut-off point of these two tests recommended in previous studies. The MMSE score was bounded by a general cut-off point of 23/24 (score considered “positive”/“negative”) for dementia [24], and for MOCA, a cut-off score of 25/26 was used for MCI [25]. Among participants with less than or equal to 12 years of education, one point was added to their total MOCA score (if < 30) to correct for education effects [25]. Participants with a MMSE score ≤ 23 or a MOCA score ≤ 25 were regarded as having cognitive impairment.

### 2.3. Data Collection

Data regarding demographic characteristics, medical history, and laboratory tests were collected from the paper-based medical records. Data were collected from clinical data of hospitalized patients. Two investigators (ZSL and LXL) independently assessed the data to ensure accuracy. The following baseline variables were obtained: age, sex, body mass index (BMI), smoking history, drinking history, diabetic course, education level (poor education: 6–12 years, high education: ≥ 12 years), diabetic macroangiopathy, diabetic microangiopathy, and complications (any chronic diseases except for those in the exclusion criteria). Diabetic macroangiopathy was defined as coronary heart disease, stroke, transient ischaemic attack, peripheral artery disease, or heart failure. Diabetic microangiopathy was defined as diabetic retinopathy, nephropathy, and peripheral neuropathy. The laboratory data collected included total cholesterol (TC), low-density lipoprotein cholesterin (LDL-C), glycosylated hemoglobin A1C (HbA1C), and P-tau181 levels. Fasting blood samples were collected after at least 10 h overnight and analysed for the biochemical measurements. TC and LDL were tested by an autoanalyzer (Hitachi 7600, Ltd, Tokyo, Japan). HbA1C was measured by high-performance liquid chromatography (HPLC) system (model HLC-723 G7; Tosoh Corporation, Tokyo, Japan). Plasma P-tau181 was measured by the Simoa HD-1 (Quanterix, Billerica, Massachusetts, United States).

### 2.4. Statistical Analysis

All statistical analyses were performed using IBM SPSS Statistics (version 20.0, IBM Corp., Armonk, New York, United States). The normality Kolmogorov–Smirnov tests were employed to analyse the data distributions. Normally distributed variables were expressed as the mean ± standard deviation and analysed using Student's *t*-test. Variables with skewed distributions were expressed as median (interquartile range, IQR) and analysed using the Mann–Whitney *U* test. Categorical variables are presented as numbers and percentages and were analysed using the chi-square test. Pearson's correlation analysis was performed to assess the association between P-tau181, MOCA, and MMSE scores. Binary logistic regression analysis was used to calculate the odds ratios (OR) and 95% confidence intervals (CIs) for cognitive impairment in patients with T2D. All variables significantly associated with cognitive impairment in the univariate analysis were further assessed by stepwise multivariate analysis (forward) to identify independent risk factors. Then, receiver operating characteristic (ROC) curve analysis was performed to test the ability of candidate variables to diagnose cognitive dysfunction in patients with T2D, and Youden's indices were calculated to select cut-offs with maximal sensitivity and specificity. A prediction model of cognitive impairment was established using a nomogram, which was performed using R version 4.0.2 (The R Foundation, Vienna, Austria). Statistical significance was set at *p* < 0.05.

## 3. Results

### 3.1. Clinical Characteristics of the Training and Validation Cohorts

A total of 667 patients with diabetes were included in the present study, with 379 and 288 cases in the training and validation cohort, respectively. General characteristics of the training and validation cohorts are summarised in Table 1 and Table S1, respectively.

In the training cohort, 181 of the 379 patients were diagnosed with cognitive impairment. Compared with patients with normal cognition, patients with cognitive impairment were older (63.2 ± 9.2 vs. 54.8 ± 8.4, *p* < 0.001), were more frequently female (43.6% vs. 30.8%, *p* = 0.01), and more frequently had poor education (69.1% vs. 39.4%, *p* < 0.001). Patients with cognitive impairment also had a longer duration of diabetes (9.0 [4.5–16.0] vs. 6.0 [1.8–12.0], *p* < 0.01), a higher proportion of complications (*p* < 0.01), and a higher incidence of diabetic retinopathy (*p* < 0.001), diabetic nephropathy (*p* < 0.05), and diabetic peripheral neuropathy (*p* < 0.05). Regarding laboratory findings, patients with cognitive impairment had lower TC (4.1 ± 1.2 vs. 4.4 ± 1.2, *p* < 0.05) and higher P-tau181 levels (13.3 [10.5–18.7] vs. 10.0 [8.0–13.0], *p* < 0.001). BMI, blood pressure, smoking time, drinking time, diabetic macroangiopathy, low-density lipoprotein, and HbA1C level were not significantly different between patients with normal cognition and those with cognitive impairment (Table 1).

One hundred and forty of the 288 cases were diagnosed with cognitive impairment in the validation cohort. Consistent with the training cohort, patients with cognitive impairment in the validation cohort were also older, were more frequently female, were less educated, had a longer duration of diabetes, had a higher proportion of complications, and had a higher incidence of diabetic retinopathy and diabetic nephropathy. Laboratory findings also revealed that patients with cognitive impairment had lower TC, LDL, and higher P-tau181 levels than those without cognitive impairment. BMI, blood pressure, smoking time, drinking time, diabetic peripheral neuropathy, diabetic macroangiopathy, and HbA1C levels were not significantly different between the two groups in the validation cohort (Table S1).

### 3.2. P-tau181 Levels Were Negatively Correlated With MOCA and MMSE

**Figure 1:**
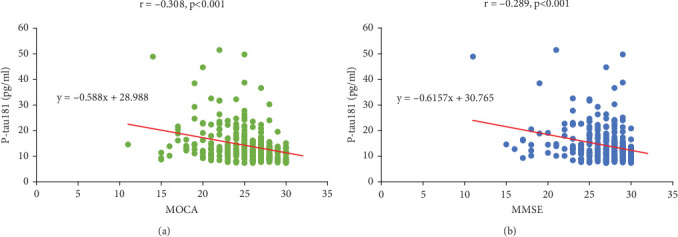
P-tau181 negatively correlates with MOCA and MMSE in T2D patients. Pearson's correlation analysis was used to assess the relationship of tau181 with (a) MOCA and (b) MMSE.

Plasma P-tau181 is a potential prognostic biomarker of AD and has attracted interest. Next, we performed a correlation analysis to investigate the relationship between plasma P-tau181 levels and the MOCA and MMSE scores. The results showed that P-tau181 levels were negatively correlated with MOCA (*r* = −0.308, *p* < 0.001) and MMSE (*r* = −0.289, *p* < 0.001) (Figure 1), indicating that higher level of P-tau181 may predict the risk of cognitive impairment.

### 3.3. Predictors of Cognitive Impairment in Diabetes Patients

Univariate and multivariate logistic regression analyses were performed to identify the risk factors for cognitive impairment in patients with T2D. Univariate analysis showed that age (OR, 1.112 [95% CI, 1.083–1.143]; *p* < 0.001), female sex (OR, 1.739 [95% CI, 1.142–2.650); *p* = 0.01), poor education (OR, 3.090 [95% CI, 2.245–5.253]; *p* < 0.001), TC (OR, 0.800 [95% CI, 0.668–0.958]; *p* < 0.05), diabetic course (OR, 1.041 [95% CI, 1.015–1.072]; *p* < 0.01), diabetic retinopathy (OR, 4.413 [95% CI, 2.699–7.216]; *p* < 0.001), diabetic nephropathy (OR, 1.588 [95% CI, 1.032–2.442]; *p* < 0.05), diabetic peripheral neuropathy (OR, 1.538 [95% CI, 1.016–2.326]; *p* < 0.05), complications (OR, 1.712 [95% CI, 1.169–2.646], *p* < 0.05), and P-tau181 (OR, 1.160 [95% CI, 1.107–1.215], *p* < 0.001) were associated with cognitive impairment in patients with diabetes (Table 2). All factors significantly associated with cognitive impairment in the univariate analysis were further assessed using stepwise multivariate analysis (forward).

Finally, four independent risk factors for diabetic cognitive impairment were identified in multivariate analysis (Table 2). Diabetic retinopathy was the strongest independent risk factor (OR, 3.923 [95% CI, 2.189–7.029]; *p* < 0.001), followed by poor education (OR, 2.283 [95% CI, 1.372–3.798]; *p* < 0.001), P-tau181 (OR, 1.137 [95% CI, 1.080–1.198]; *p* < 0.001), and age (OR, 1.096 [95% CI, 1.064–1.130]; *p* < 0.001).

### 3.4. Nomogram for Screening of Cognitive Impairment in T2D Patients and Predictive Performance

**Figure 2:**
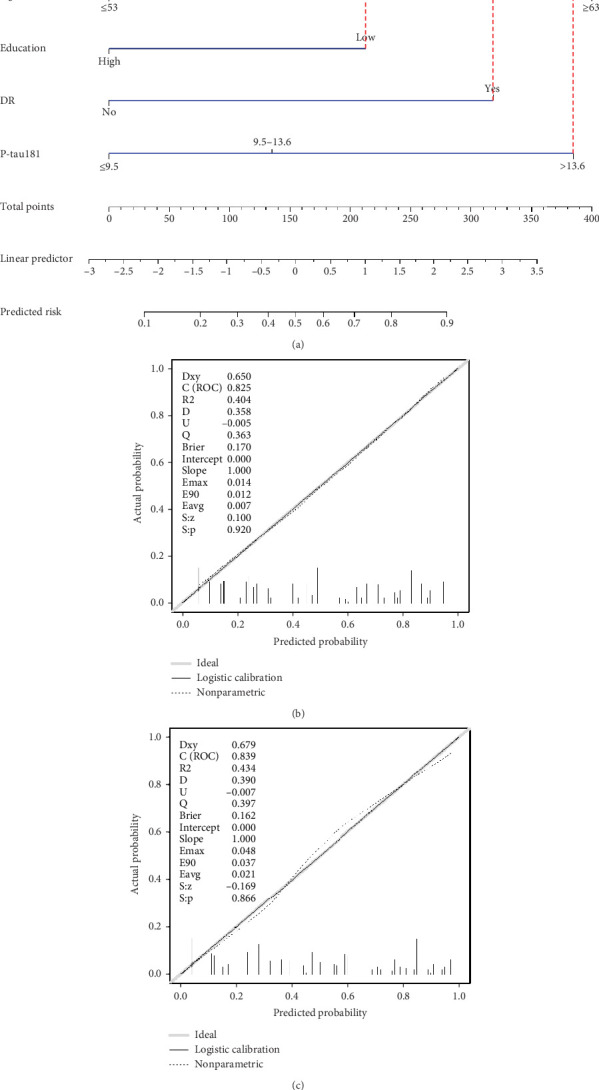
Prediction model of cognitive dysfunction in T2D patients. (a) Nomogram was established as a prediction model based on the four independent predictors of cognitive dysfunction. Calibration curves for the (b) training cohort and the (c) validation cohort were used to assess the calibration of the model.

**Figure 3:**
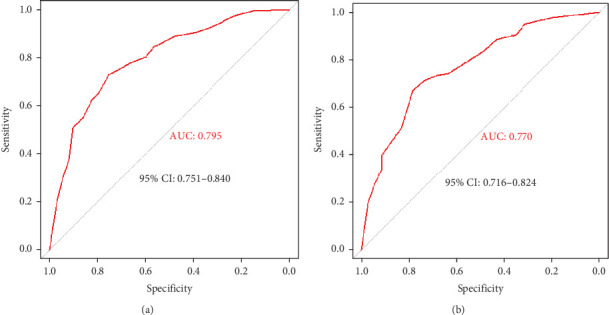
Receiver operating characteristic curves were used to analyze the discrimination ability of the nomogram-based prediction model for cognitive dysfunction in T2D patients in the (a) training cohort and the (b) validation cohort.

A screening model of cognitive impairment in patients with diabetes was established using a nomogram by incorporating four significant risk factors from the multivariate analysis (age, education, diabetic retinopathy, and P-tau181) (Figure 2a). To facilitate clinical use, we established the diabetic cognitive impairment score (DCIS). In the DCIS screening model, four factors were weighted according to their weight in the nomogram. The age was categorised into 53 or less, 54–62, and ≥ 63 according to the nomogram and weighted as 0, 39, and 62 points, respectively. Education was weighted as 53 (low education) and 0 (high education) points. Diabetic retinopathy was weighted as 80 (yes) and 0 (no) points. P-tau181 levels were categorised into 9.5 or less, 9.6–13.6, and > 13.6 according to the nomogram and weighted as 0, 34, and 96 points, respectively (Figure 2a and Table 3). Patients with poor education and diabetic retinopathy, age over 63 years, and P-tau181 > 13.6 pg/mL had a total score of 291 points, and their risk of diabetic cognitive impairment was greater than 90%. The calibration curves showed good consistency between the actual probability of cognitive impairment and the predicted probability of cognitive impairment estimated using the nomogram (Figure 2b). In the training cohort, ROC curve analysis revealed that DCIS could accurately predict cognitive impairment in diabetes patients, with an area under ROC curve (AUC) of 0.795 (95% CI, 0.751–0.840) (Figure 3a). The optimal cut-off of the screening model for the diagnosis of cognitive impairment was 139.5, with a sensitivity of 72.9% and a specificity of 75.3% in the training cohort.

### 3.5. Validation of the Screening Model

The performance of the screening model was further tested in the validation cohort. In the validation cohort, the nomogram also displayed a good *C* index for the diagnosis of cognitive impairment in patients with diabetes and a good agreement in the calibration curves (Figure 2c). In the validation cohort, the AUC of the screening model was 0.770 (95% CI, 0.750–0.866) (Figure 3b). Using a cut-off score of 139.5, the sensitivity and specificity were 67.1% and 78.4%, respectively.

## 4. Discussion

In this study, we found that P-tau181 was independently associated with diabetic cognitive impairment. Based on nomogram, we developed the DCIS, including P-tau181, age, education level, and diabetic retinopathy, to identify cognitive impairment in T2D patients. Our new screening tool is useful for clinicians to differentiate T2D patients with cognitive impairment from those with normal cognition.

Cognitive impairment caused by diabetes is often neglected because of its diverse and nonspecific clinical manifestations. The learning and memory ability of patients with cognitive impairment is often decreased, and their performance in information processing speed, attention, and executive function is reduced [26–28]. In severe cases, the quality of life is affected by an obvious decline in the ability to perform daily activities. Cognitive impairment can worsen the treatment compliance of patients with diabetes, and they are prone to severe hypoglycaemia, hyperglycaemia, and other acute complications of diabetes. Severe hypoglycaemia can aggravate cognitive impairment, and dementia can mask the clinical symptoms of hypoglycaemia or hyperglycaemia. Diabetes and cognitive impairment affect and promote each other, thereby forming a vicious circle. Therefore, early screening is of great significance.

Numerous risk factors have been associated with the occurrence and development of cognitive impairment in diabetes. These reported risk factors include non-T2D-associated and T2D-associated risk factors. Non-T2D-associated risk factors included age, gender, genetic factors, blood pressure, blood lipids, tau phosphorylation, homocysteine levels, smoking, alcohol consumption, obesity, education level, and depression [29, 30]. T2D-associated risk factors include hyperglycaemia, hypoglycaemia, glucose fluctuation, diabetes duration, insulin resistance, amyloid-beta peptide accumulation, cerebral arteriosclerosis, and microvascular complications [29, 30]. Consistent with previous studies, our study also showed that age, gender, education level, TC, diabetic course, diabetic microvascular complications, and tau phosphorylation were risk factors for diabetic cognitive impairment in the univariate analysis. However, in the subsequent stepwise multivariate analysis, we found that only age, education level, diabetic retinopathy, and P-tau181 level were independent risk factors for DCI. The difference in risk factors among studies may be explained by the fact that there are many different factors involved, but the effects of each factor are weak [1]. Among these factors, blood glucose control has received extensive attention. Brain and neural tissues mainly depend on glucose as energy substrate in physiological states, and therefore, any alterations in carbohydrate metabolism will directly affect cerebral function, including cognition. Individuals with poor glycaemic control performed poorly on memory tests. Some evidence suggests that higher HbA1C levels are associated with diabetes-related cognitive decline, but the intensity of this relationship is low [31]. This may be because cognitive impairment caused by hyperglycaemia is a lengthy process, and HbA1C level cannot reflect such a long-term glycaemic control. Our study did not show a high HbA1C level as a risk factor; instead, we found that diabetic retinopathy was a robust risk factor. Consistent with our findings, the association between diabetic retinopathy and cognitive impairment has been well documented. A recent meta-analysis of 25 studies (17 cross-sectional and 8 longitudinal studies) with a total of 1,963,914 subjects observed that among the cross-sectional studies, the pooled ORs of cognitive impairment were 1.48 in subjects with diabetic retinopathy and, among the longitudinal studies, the pooled RRs of cognitive impairment in subjects with diabetic retinopathy were 1.35 [32]. The association between diabetic retinopathy and cognitive impairment is possibly because the retina is an ontogenetic brain-derived tissue, and as ontogenically linked organs, the brain and retina often show shared pathologies [33]. Therefore, diabetic retinopathy could potentially predict the development of cognitive decline.

We found that a high P-tau181 level was a robust risk factor. It is reported that CSF P-tau levels are significantly higher in patients with MCI; therefore, P-tau can be used as an early marker of cognitive disorders [34]. Compared with healthy controls, patients with T2DM show significantly increased levels of cerebrospinal fluid (CSF) P-tau [35]. However, CSF examination is invasive and limited in routine clinical use. Plasma P-tau181 levels have been shown to be significantly positively correlated with both P-tau181 and tau-PET in CSF, and it appears to be a promising tool for rapid diagnosis of cognitive impairment in patients with diabetes. The P-tau181 levels have shown a significantly negative correlation with the MMSE and MOCA scores in our study. The increase in CSF tau reflects changes in axonal degeneration and NFTs in patients with AD, following the release of tau protein into the CSF [36]. Increased levels of CSF P-tau reflect pathophysiological changes in AD and specifically suggest formation of NFTs. Compared to CSF P-tau, plasma P-tau181 is a potential noninvasive diagnostic and prognostic biomarker of cognitive disorders [37], which can improve the accuracy of the clinical diagnosis of AD [38]. Plasma P-tau181 was increased at the MCI and dementia stages [37]. There was a significant negative correlation between the increase of plasma p-tau181 and structural changes in widespread brain regions [39]. Consistent with these studies, we also found that plasma P-tau181 levels increased in diabetic patients with cognitive impairment.

Other risk factors in our study include age and education level, both of which have been proven to be associated with cognitive impairment. The risk of cognitive impairment in individuals with low education is approximately two times higher than that in individuals with high education. Numerous studies have shown a negative correlation between education and the risk of cognitive impairment [40, 41]. A potential explanation is that people with higher education have a greater cognitive reserve which refers to the ability of the brain to cope with neuropathology or injury and has been proposed to reduce the risk of dementia and cognitive decline [42]. Age is another risk factor. It is well known that cognitive impairment is positively correlated with age, and the prevalence of dementia increases with aging. Most research on cognitive impairment focuses on people over age 65. In fact, researches have revealed that cognitive decline in diabetes is not limited to older people, but may begin to appear in middle age [43–45]. This supports our results. We observed that a part of middle-aged patients with diabetes have already developed cognitive impairment. We also found that patients with cognitive impairment are older than those with normal cognition, indicating an increased risk of cognitive impairment with age.

In the current study, by incorporating these four risk factors into DCIS, we established a useful clinical screening tool for patients with cognitive impairment. At present, the diagnosis of dementia is mainly based on clinicians' suspicion of patients' symptoms, rather than routine formal screening. Up to 29%–76% of patients with dementia or possible dementia remain undiagnosed [46, 47]; this excludes patients with MCI. Screening tests for cognitive impairment in clinical settings generally include a variety of neuropsychological assessment tools. Radiological examinations, blood tests, and CSF tests are not currently used as screening tests but are usually used to confirm the diagnosis and subtype of dementia after a positive screening result. However, neuropsychological evaluation is difficult and time-consuming for nonneurologists, which limits the routine screening of cognitive impairment. The screening tools we developed can be easily used in clinics, and the use of a few variables and visual scoring of the nomogram ensures that DCIS is simple and convenient. In addition, we converted the nomogram into a simple score to facilitate its wide clinical application. Patients with positive screening results can be further evaluated by neurologists.

The current study had several limitations. First, the DCIS was developed for an Asian cohort, and whether it can be extended to other populations requires further research. Second, many elderly patients were excluded from the study because of illiteracy, resulting in selection bias. Third, the DCIS model cannot distinguish between types of cognitive impairment, such as MCI, AD, and vascular dementia, and we will further study how to accurately identify specific types of cognitive impairment in diabetes. Moreover, in the validation cohort, the screening model has a low sensitivity of 67.1%. The reason for the low sensitivity may be insufficient sample size, class imbalance, lack of included indicators, etc.

## 5. Conclusions

In conclusion, we found that P-tau 181 was an independent risk factor for diabetic cognitive impairment. We developed a scoring system DCIS based on four risk factors including P-tau181, age, education level, and diabetic retinopathy, to reliably screen diabetes patients with cognitive impairment. We determined that the optimal cut-off point of DCIS for identifying cognitive impairment in T2D patients was 139.5. Our findings have important clinical implications. The DCIS score may be widely used for identification and subsequent management of cognitive impairment in T2D patients. In the future, it is necessary to explore more effective prediction models in large and various populations.

## Figures and Tables

**Table 1:** Baseline characteristics of diabetes patients in the training cohort.

	**Total**	**Normal**	**Cognitive dysfunction**	**p**
*N*	379	198 (52.2%)	181 (47.8%)	
Age (years)	58.8 ± 9.7	54.8 ± 8.4	63.2 ± 9.2	< 0.001
Female	140 (36.9%)	61 (30.8%)	79 (43.6%)	0.010
BMI (kg/m^2^)	23.9 ± 4.1	23.6 ± 3.8	24.1 ± 5.1	0.621
SBP (mmHg)	128 ± 19	128 ± 19	128 ± 20	0.964
DBP (mmHg)	80 ± 11	80 ± 10	80 ± 11	0.772
Smoke (yes)	124 (32.8%)	73 (37.1%)	51 (28.2%)	0.066
Drink (yes)	80 (21.1%)	47 (23.7%)	33 (18.2%)	0.190
Poor education	203 (53.6%)	78 (39.4%)	125 (69.1%)	< 0.001
TC (mmol/L)	4.3 ± 1.2	4.4 ± 1.2	4.1 ± 1.2	0.013
LDL (mmol/L)	2.5 ± 0.9	2.6 ± 0.9	2.4 ± 1.0	0.057
HbA1C (%)	8.9 ± 2.4	8.9 ± 2.3	8.8 ± 2.4	0.764
Diabetic course (years)	8.0 (3.0–15.0)	6.0 (1.8–12.0)	9.0 (4.5–16.0)	0.002
Diabetic retinopathy	107 (28.2%)	29 (14.6%)	78 (43.1%)	< 0.001
Diabetic nephropathy	126 (33.3%)	56 (28.4%)	70 (38.7%)	0.035
Diabetic peripheral neuropathy	224 (59.3%)	107 (54.3%)	117 (64.6%)	0.041
Macrovascular complication	279 (73.8%)	144 (73.1%)	135 (74.6%)	0.742
Complications	198 (52.4%)	90 (45.7%)	108 (59.7%)	0.007
MOCA	26 (23–28)	27 (26–29)	23 (21–25)	< 0.001
MMSE	28 (26–29)	29 (28–30)	26 (24–27)	< 0.001
P-tau181 (pg/mL)	11.7 (8.7–15.1)	10.0 (8.0–13.0)	13.3 (10.5–18.7)	< 0.001

*Note:* Data are presented as means ± SD, median (interquartile range), and proportion (%).

Abbreviations: BMI, body mass index; DBP, diastolic blood pressure; LDL, low-density lipoprotein; SBP, systolic blood pressure; TC, total cholesterol.

**Table 2:** Logistic regression analysis of predictors of cognitive dysfunction in diabetes patients.

**Variable**	**Univariate analysis**		**Stepwise multivariate analysis (forward)**	
	**OR (95% CI)**	**p**	**OR (95% CI)**	**p**
Age (years)	1.112 (1.083–1.143)	< 0.001	1.096 (1.064–1.130)	< 0.001
Female	1.739 (1.142–2.650)	0.010	—	—
Poor education	3.090 (2.245–5.253)	< 0.001	2.283 (1.372–3.798)	< 0.001
TC (mmol/L)	0.800 (0.668–0.958)	0.015	—	—
Diabetic course (years)	1.041 (1.015–1.072)	0.004	—	—
Diabetic retinopathy	4.413 (2.699–7.216)	< 0.001	3.923 (2.189–7.029)	< 0.001
Diabetic nephropathy	1.588 (1.032–2.442)	0.035	—	—
Diabetic peripheral neuropathy	1.538 (1.016–2.326)	0.042	—	—
Complications	1.712 (1.169–2.646)	0.012	—	—
P-tau181 (pg/mL)	1.160 (1.107–1.215)	< 0.001	1.137 (1.080–1.198)	< 0.001

*Note:* Data are expressed as odds ratio (OR) (95% CI).

Abbreviation: TC, total cholesterol.

**Table 3:** Nomogram-based preoperative score: Four-variable model.

**Variable**	**Category**	**Points**
Age (years)	≤ 53	0
	54–62	39
	≥ 63	62

Education	High	0
	Low	53

DR	No	0
	Yes	80

P-Tau181 (pg/mL)	≤ 9.5	0
	9.6–13.6	34
	> 13.6	96

*Note:* Possible score ranges from 0 to 291.

Abbreviation: DR, diabetic retinopathy.

## Data Availability

Data are available on reasonable request.

## Supporting Information

Supporting Information 1 Supplementary Table S1. Baseline characteristics of diabetes patients in the validation cohort.

Supporting Information 2 Supplementary Figure S1. Flowchart of study participants.
