# Supplementary material for: The Diabetic Cognitive Impairment Score for Early Screening of Cognitive Impairment in Type 2 Diabetes Patients
Source: J Diabetes Res. 2025 Apr 16;2025:8029913. doi: 10.1155/jdr/8029913 (PMC12017955; doi:10.1155/jdr/8029913)
Supplement: Supporting Information 1 — Supplementary Table S1. Baseline characteristics of diabetes patients in the validation cohort. [file 8029913.f1.docx]

Supplemental Table S1. Baseline characteristics of diabetes patients in the validation cohort.

|  | Total | Normal | cognitive dysfunction | P |
| --- | --- | --- | --- | --- |
| N | 288 | 148 | 140 |  |
| **Age (years)** | 57.1±10.1 | 53.9±8.6 | 60.5±10.4 | **<0.001** |
| **Female** | 108 (37.5%) | 47 (31.8%) | 61 (43.6%) | **0.038** |
| BMI (kg/m^2^) | 23.6±3.7 | 22.7±3.1 | 24.2±4.5 | 0.131 |
| SBP (mmHg) | 126±21 | 122±20 | 129±24 | 0.209 |
| DBP (mmHg) | 83±14 | 80±15 | 85±16 | 0.176 |
| Smoke (yes) | 85 (29.5%) | 50 (33.8%) | 35 (25.0%) | 0.102 |
| Drink (yes) | 57 (19.8%) | 30 (20.3%) | 27 (19.3%) | 0.834 |
| **Poor education** | 130 (45.1%) | 41 (27.7%) | 89 (63.6%) | **<0.001** |
| **TC (mmol/L)** | 4.3±1.2 | 4.5±1.2 | 4.1±1.2 | **0.001** |
| **LDL (mmol/L)** | 2.5±0.9 | 2.7±1.0 | 2.4±0.9 | **0.004** |
| HbA1C (%) | 9.0±2.5 | 9.0±2.2 | 9.1±2.8 | 0.871 |
| **Diabetic course (years)** | 7.0 (4.0-12.0) | 6.0 (3.0-11.0) | 8.0 (5.0-15.0) | **0.014** |
| **Diabetic retinopathy** | 77 (26.7%) | 20 (13.5%) | 57 (40.7%) | **<0.001** |
| **Diabetic nephropathy** | 98 (34.0%) | 41 (27.7%) | 57 (40.7%) | **0.020** |
| Diabetic peripheral neuropathy | 156 (54.2%) | 72 (48.6%) | 84 (60.0%) | 0.053 |
| Macrovascular complication | 199 (69.1%) | 102 (68.9%) | 97 (69.3%) | 0.946 |
| **Complications** | 153 (53.1%) | 68 (45.9%) | 85 (60.7%) | **0.012** |
| **MoCA** | 26 (23-27) | 27 (26-28) | 23 (21-25) | **<0.001** |
| **MMSE** | 28 (25-29) | 28 (28-29) | 25 (23-27) | **<0.001** |
| **P-tau181 (pg/ml)** | 12.0 (8.8-14.7) | 10.5 (8.2-13.5) | 13.2 (11.0-16.7) | **<0.001** |

Data are presented as means ± SD, median (interquartile range), and proportion (%). BMI: body mass index. SBP: systolic blood pressure. DBP: diastolic blood pressure. TC: total cholesterol. LDL:low-density lipoprotein.
