# Supplementary figures and images for: The Diabetic Cognitive Impairment Score for Early Screening of Cognitive Impairment in Type 2 Diabetes Patients
Source: J Diabetes Res. 2025 Apr 16;2025:8029913. doi: 10.1155/jdr/8029913 (PMC12017955; doi:10.1155/jdr/8029913)

Figure S1. Flowchart of study participants.


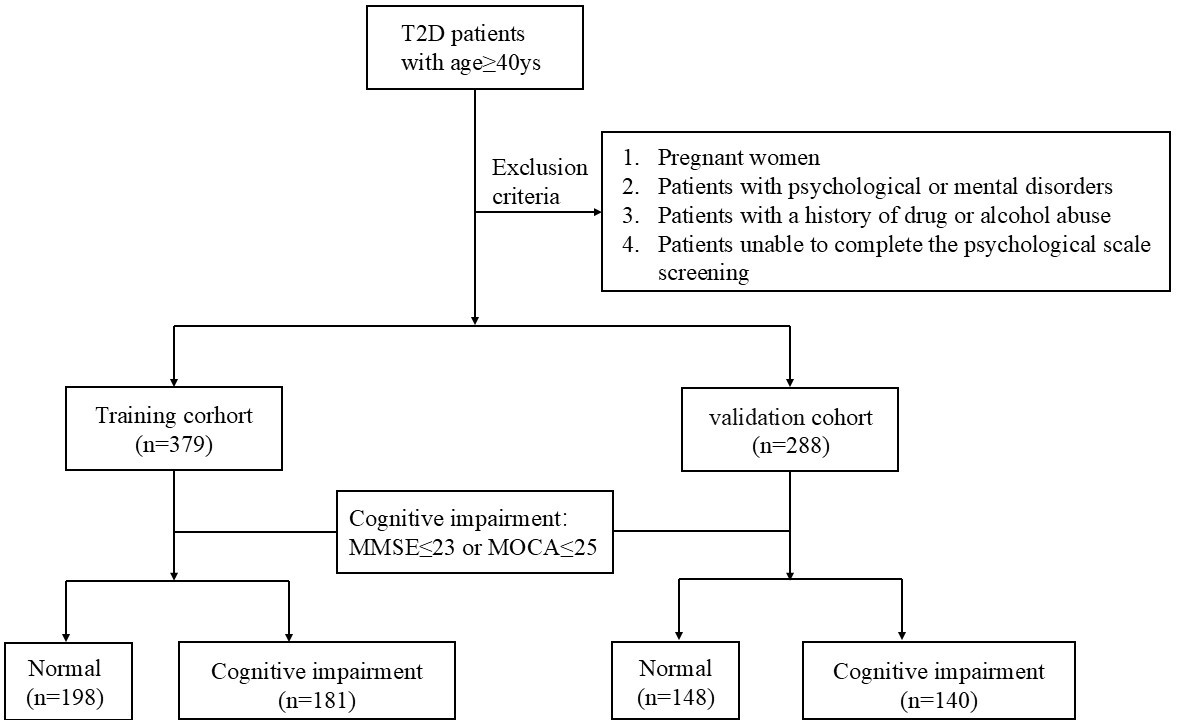

Supplement: Supporting Information 2 — Supplementary Figure S1. Flowchart of study participants. [file 8029913.f2.docx]
